# MutDock: A computational docking approach for fixed-backbone protein scaffold design

**DOI:** 10.3389/fmolb.2022.933400

**Published:** 2022-08-29

**Authors:** Varun M. Chauhan, Robert J. Pantazes

**Affiliations:** Chemical Engineering Department, Auburn University, Auburn, AL, United States

**Keywords:** protein docking, protein scaffold, force field, hydrogen bonds, binding energy

## Abstract

Despite the successes of antibodies as therapeutic binding proteins, they still face production and design challenges. Alternative binding scaffolds of smaller size have been developed to overcome these issues. A subset of these alternative scaffolds recognizes target molecules through mutations to a set of surface resides, which does not alter their backbone structures. While the computational design of antibodies for target epitopes has been explored in depth, the same has not been done for alternative scaffolds. The commonly used dock-and-mutate approach for binding proteins, including antibodies, is limited because it uses a constant sequence and structure representation of the scaffold. Docking fixed-backbone scaffolds with a varied group of surface amino acids increases the chances of identifying superior starting poses that can be improved with subsequent mutations. In this work, we have developed MutDock, a novel computational approach that simultaneously docks and mutates fixed backbone scaffolds for binding a target epitope by identifying a minimum number of hydrogen bonds. The approach is broadly divided into two steps. The first step uses pairwise distance alignment of hydrogen bond-forming areas of scaffold residues and compatible epitope atoms. This step considers both native and mutated rotamers of scaffold residues. The second step mutates clashing variable interface residues and thermodynamically unfavorable residues to create additional strong interactions. MutDock was used to dock two scaffolds, namely, Affibodies and DARPins, with ten randomly selected antigens. The energies of the docked poses were minimized and binding energies were compared with docked poses from ZDOCK and HADDOCK. The top MutDock poses consisted of higher and comparable binding energies than the top ZDOCK and HADDOCK poses, respectively. This work contributes to the discovery of novel binders based on smaller-sized, fixed-backbone protein scaffolds.

## Introduction

Binding proteins are an integral part of innumerable biological processes. Their roles include performing catalysis by binding to substrates as enzymes (Vaissier Welborn and Head-Gordon, 2019), transporting ligands across cell membranes as carrier and channel proteins (Barabote et al., 2006), and antibodies binding to foreign antigens to tag them for destruction by vertebrate immune systems (Lu et al., 2020). Antibodies have become the most important type of binding protein, with a global therapeutic market value of over $100 billion (Lu et al., 2020), and their structures have been extensively studied (Ducancel and Muller, 2012). The binding domains of antibodies consist of two regions: a scaffold-like, highly-conserved framework region and hypervariable binding loops (i.e., complementarity determining regions (CDRs)) that interact with antigens (Pantazes and Maranas, 2010). The design and engineering of antibodies are primarily carried out *via* experimental methods such as hybridoma technology or synthetic library surface display (Almagro et al., 2019).

While successful, there are several drawbacks to the experimental design and generation of antibodies: the presence of multiple chains, disulfide bonds, and glycans complicates their lab-based generation, purification, and formulation (Gebauer and Skerra, 2020), and experimental approaches are not capable of generating binders that target a specific epitope without extensive screening and a measure of luck. Furthermore, these methods tend to be expensive and time-consuming (Norman et al., 2020). To overcome these challenges, a number of computational methods have been developed for the epitope-specific design of antibodies, which can later be improved through experimental means (Pantazes and Maranas, 2010; Lapidoth et al., 2015; Liu et al., 2017; Adolf-Bryfogle et al., 2018; Chowdhury et al., 2018; Nimrod et al., 2018). A common feature of these computational approaches is that they take advantage of the structural features of antibodies and develop initial designs by swapping CDRs to find an antibody with a shape that matches the target antigen.

Although antibodies have been very successful, they may not be the best choice for all contexts where protein binding is needed. An alternative to their use is smaller-sized protein domains, including Knottins (Moore and Cochran, 2012), Kunitz domains (Hosse et al., 2006), Fynomers (Bertschinger et al., 2007), and Fibronectin domains (Koide et al., 1998), among others. The benefits of using smaller-sized scaffolds over antibodies include better thermostability, higher tumor penetration, lower cost of production, and decreased chance of denaturation (Gilbreth and Koide, 2012; Stern et al., 2013; Richards, 2018). Although not as common as antibodies, these smaller scaffolds have seen success as therapeutics: drugs developed using Kunitz domains and Knottins have been approved by the FDA, while other alternative scaffold-based drugs are in different phases of clinical trials (Simeon and Chen, 2018).

**FIGURE 1:**
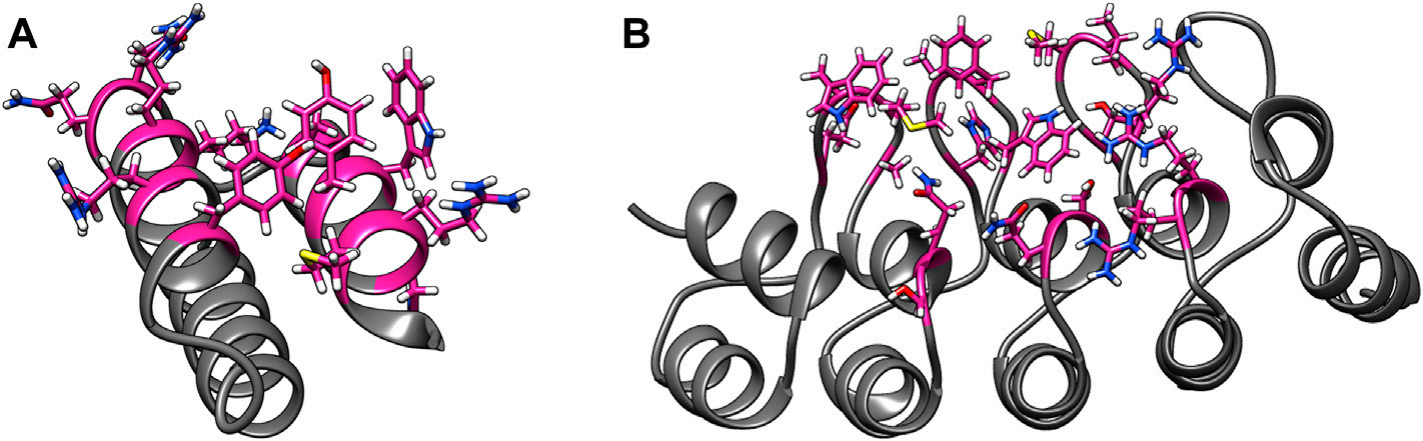
Examples of alternative binding scaffolds. Depicted are **(A)** Affibody (PDB: 3MZW) and **(B)** DARPin (PDB: 6FPA) structures. Their variable residues that mutate to bind target proteins are colored in pink.

Some of these alternative scaffolds bind to target molecules with modular loops comparable to the CDRs of antibodies, and such proteins can be designed with computational protocols similar to those developed for antibodies. However, binding for a subclass of these alternative scaffolds is governed by point mutations to surface residues in highly stable secondary structures, and these mutations do not alter the proteins’ structures. Examples of such binding proteins include affibodies and designed ankyrin repeats (DARPins) (Alsultan et al., 2016). An affibody consists of 58 amino acids and is arranged in a three alpha–helix bundle framework. The design of antigen-specific affibodies has been primarily carried out through combinatorial mutations of 13 surface residues on two helices (Ståhl et al., 2017). Affibodies have been designed to bind over 40 antigens and the HER-2 binding affibody ABY-025 has reached phase 2/3 clinical trial (Altunay et al., 2021). A DARPin molecule consists of 33 residue long motifs that are typically repeated two to four times, along with N and C terminal motifs. Similar to affibodies, binders are designed by mutating six residues in each motif barring the terminal motifs (Shilova and Deyev, 2019). Abicipar, a VEGF-A binding therapeutic DARPin drug, has reached phase III clinical trial (Simeon and Chen, 2018). The structures and variable residues of affibodies and DARPins are shown in Figure 1.

Computational methods to design affibodies and DARPins require different approaches than those for antibodies due to their lack of loops analogous to CDRs. One strategy is to use a docking program (Chen et al., 2003; Dominguez et al., 2003; Comeau et al., 2004; Lyskov and Gray, 2008; Torchala et al., 2013) to create an initial complex followed by iterative cycles of point mutations (Pantazes et al., 2015; Adolf-Bryfogle et al., 2018; Nimrod et al., 2018). This approach is analogous to and inspired by the affinity maturation process of antibodies by the immune system (Teng and Papavasiliou, 2007) and the experimental directed evolution protocol (Arslan et al., 2019). Various docking approaches have been developed over the past 2 decades. ZDOCK uses stepwise movements and rotations of rigid body representation of the ligand around the receptor and uses fast Fourier transforms to quickly identify poses with good shape complementarity features. Poses are then ranked based on energy potentials (Chen et al., 2003). Other approaches like ClusPro use ZDOCK for good quality pose identification followed by further pose refinement and binding energy evaluations (Comeau et al., 2004). RosettaDock uses a coarse-grained rigid body Monte Carlo search for high-scoring poses, which are later refined through local docking accompanied with side chain and backbone packing and energy minimizations. The Rosetta energy function is used in the Monte Carlo search and to rank poses (Lyskov and Gray, 2008). Swarmdock, a population-based metaheuristic approach, starts with a group of random initial poses and uses a particle swarm optimization algorithm to minimize electrostatic and van der Waals (vdW) potentials between two proteins (Torchala et al., 2013). HADDOCK uses a combination of rigid body energy minimizations of randomly generated starting poses and flexible energy minimizations of the best 1,000 initial complexes (Dominguez et al., 2003).

Docking approaches use rigid-structure and/or fixed-sequence representations of scaffolds since the original purpose of such tools is to predict native binding orientations. While appropriate for replicating native complexes, this feature imposes limitations for designing binding proteins because it will reject protein poses with clashes between native side chains that could be rectified through mutations. A design approach that can dock scaffolds while mutating residues would be likely to identify higher quality complexes than methods that cannot.

One strategy to do this would be to dock proteins in a manner where they form strong interactions which are known to be abundant in protein–protein binding interfaces, such as hydrogen bonds (H-bonds) and hydrophobic interactions. The Baker lab has developed RIFdock, an approach that docks proteins to a ligand or another protein while simultaneously making mutations. RIFdock performs this by docking individual amino acids to the epitope, generating a large library of reverse rotamers for the well-docked amino acids and identifying scaffold positions that can hold multiple reverse rotamers (Dou et al., 2018). Cao et al. designed SARS-CoV-2 binding miniproteins by initially scanning a library of 19,000 miniproteins through RIFdock against the ACE-2 binding epitope of the receptor-binding domain (RBD). High-quality poses were then experimentally affinity matured to bind with picomolar affinity with the target ACE-2 binding epitope (Longxing et al., 2020). Similar to other docking approaches like ZDOCK and HADDOCK, RIFdock makes use of grid-based movements of the scaffold around the target protein. As of the submission of this work, a detailed description of the RIFdock methodology is not available in a peer-reviewed article. Furthermore, RIFdock is not available in the Rosetta Commons.

Here, the algorithm and initial evaluation of MutDock, a novel mutation-based docking approach, are described. Instead of translating and rotating a protein scaffold in 6D steps around the target, MutDock uses pairwise distance matching of H-bonding regions around the variable paratope and epitope to identify mutated scaffold-target poses, making multiple H-bonds in a single step. MutDock initially identifies H-bond-forming regions around the paratope and epitope and subsequently searches for different combinations of interactions with compatible geometries that constitute non-clashing docked poses.

## Materials and methods

MutDock has been developed around two major goals: 1) introducing mutations simultaneously to docking using a single geometry alignment step and 2) designing binding proteins based on known beneficial structural elements. Instead of using force field–guided energy minimizations and scoring functions to assess randomly docked poses like conventional docking tools such as RosettaDock and HADDOCK, MutDock uses the known structural features of strong interactions such as H-bonds to guide pose identification and rotamer selection during docking. Similar to ZDOCK, where the generated poses are scored in the final step, in this work, force fields are used to evaluate MutDock’s predicted poses but not in their initial identification.

**FIGURE 2:**
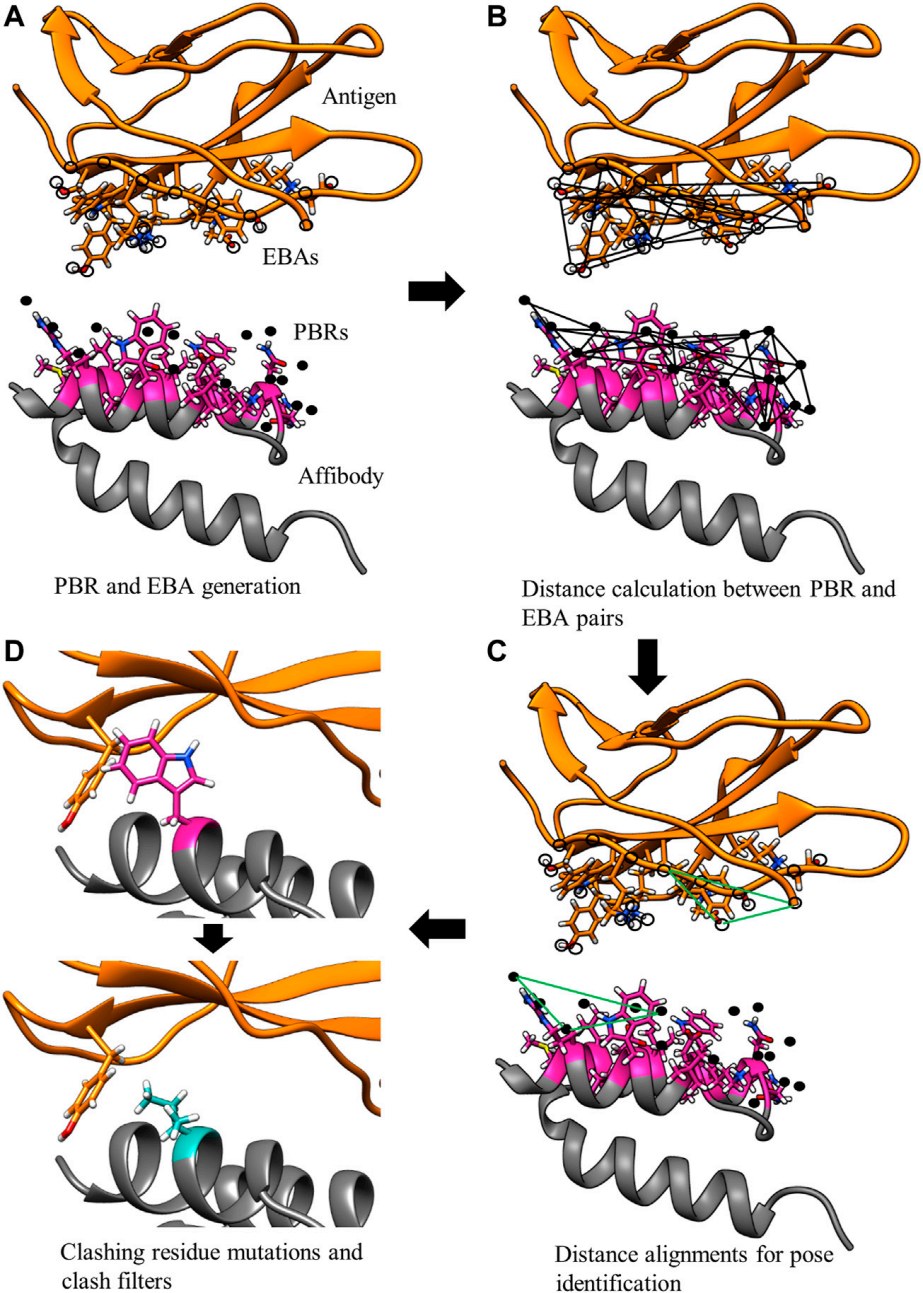
MutDock workflow. MutDock can be divided into two main steps: pose identification (panels A–C) and pose validation (panel D). Step **(A)** PBRs are identified for all paratope residues and all other rotamers of variable residues (shown in pink). Similarly, EBAs are identified for all epitope residues. Step **(B)** Pairwise distance calculations within the sets of PBRs and EBAs. Step **(C)** Pairwise distance matching between PBR pairs and EBA pairs to identify groups of compatible low entropy interactions. Step **(D)** Each pose from Step C is passed through steric clash filters, and clashing variable side chains are mutated.

The MutDock approach can be divided into two primary steps: 1) identifying docked poses with H-bonds formed by native and/or mutated residues and 2) mutating clashing side chains using a feature-based approach. This algorithm is depicted in Figure 2, with panels A, B, and C corresponding to step 1 and panel D corresponding to step 2.

### Docking

Docking with MutDock can be further subdivided into two steps: pose identification and pose validation. The pose identification step generates antigen-scaffold poses with unique sets of H-bonds, while the pose validation step checks for steric compatibility, bond formation, and solution uniqueness. Necessary information for docking includes the structures of the binding and target proteins, which residues in the binding protein are intended to interact with the target protein (i.e., the paratope), which paratope residues are mutable, and which residues in the target protein are intended to interact with the paratope (i.e., the epitope).

In the first step of pose identification, spatial coordinates that can be occupied by compatible epitope atoms for the formation of an H-bond are identified for each paratope residue. These coordinates for binding interactions, referred to as Paratope Binding Regions (PBRs), consist of one atom and three spatial positions. The atom is either hydrogen, if the interaction is an H-bond from the paratope to the epitope, or an H-bond acceptor, if the H-bond forms from the epitope to the paratope. The primary point of the PBR is the ideal position of an atom in the epitope to form an H-bond with the PBR’s atom. The position of the primary point lies 1.25 Å from the PBR’s atom on the vector determined by the atom and an antecedent point. The possible PBR atoms and corresponding antecedent points are listed in Table 1. The third spatial position of a PBR is the secondary point, 1 Å further along the vector from the primary point, which is used for ensuring the designed H-bonds have appropriate orientations (e.g., avoiding the formation of H-bonds with acute angles).

**TABLE 1:** Polar atom and their antecedent atom names considered for PBR and EBA identification. Atom names follow the CHARMM PDB atom naming.

Amino acid	PBR atom	Antecedent point
Backbone	O	C
Backbone	OT1	C
Backbone	OT2	C
Backbone	HN	N
Backbone	HN1	N
Backbone	HN2	N
Backbone	HN3	N
ARG	HE	NE
ARG	HH11	NH1
ARG	HH12	NH1
ARG	HH21	NH2
ARG	HH22	NH2
LYS	HZ1	NZ
LYS	HZ2	NZ
LYS	HZ3	NZ
ASP	OD1	CG
ASP	OD2	CG
GLU	OE1	CD
GLU	OE2	CD
SER	HG1	OG
SER	OG	Midpoint of HG1 and CB
THR	HG1	OG1
THR	OG1	Midpoint of HG1 and CB
TYR	HH	OH
TYR	OH	Midpoint of HH and CZ
ASN	OD1	CG
ASN	HD21	ND2
ASN	HD22	ND2
GLN	OE1	CD
GLN	HE21	NE2
GLN	HE22	NE2
HIS	HD1	ND1
HIS	NE2	Midpoint of CD2 and CE1
TRP	HE1	NE1

The pose identification step of MutDock is focused on finding complexes with many H-bonds because they are prevalent in naturally occurring binding interfaces. PBRs are also identified for rotamers of polar amino acids for each mutable paratope residue. As listed in Table 2, the mutable paratope residues are only allowed to change into polar residues to facilitate this search for favorable H-bonds. The rotamers used in MutDock were obtained from the Dunbrack rotamer library (Dunbrack and Cohen, 1997), and a maximum of five structurally diverse rotamers for each possible mutation were used to limit the final solution set diversity.

**TABLE 2:** Amino acid types allowed to form H-bonds in the MutDock approach.

Scaffold	Antigen
Backbone	Backbone
Backbone	All
All except ARG, LYS, and GLN	Backbone
ARG and LYS *	ASP and GLU
ASP and GLU	ARG and LYS
HIS, ASN, ASP, TYR, SER, THR, and TRP **	HIS, ASN, ASP, TYR, SER, THR, and TRP

^*^ Only one per solution.

^**^ No H-bonds allowed between ASN and ASP since they contain multiple polar groups.

Once the PBRs are identified, the epitope residues are scanned for polar atoms capable of forming H-bonds with the scaffold in MutDock’s second step of pose identification. Initially, epitope residues that have non-zero solvent accessible surface areas (SASA) are collected to avoid including buried atoms in subsequent calculations. For each solvent accessible epitope residue, epitope-binding atoms (EBAs) are identified. An EBA consists of two points: a primary point that is a polar hydrogen atom or an H-bond acceptor and a secondary point that is analogous to the antecedent points of PBRs.

**FIGURE 3:**
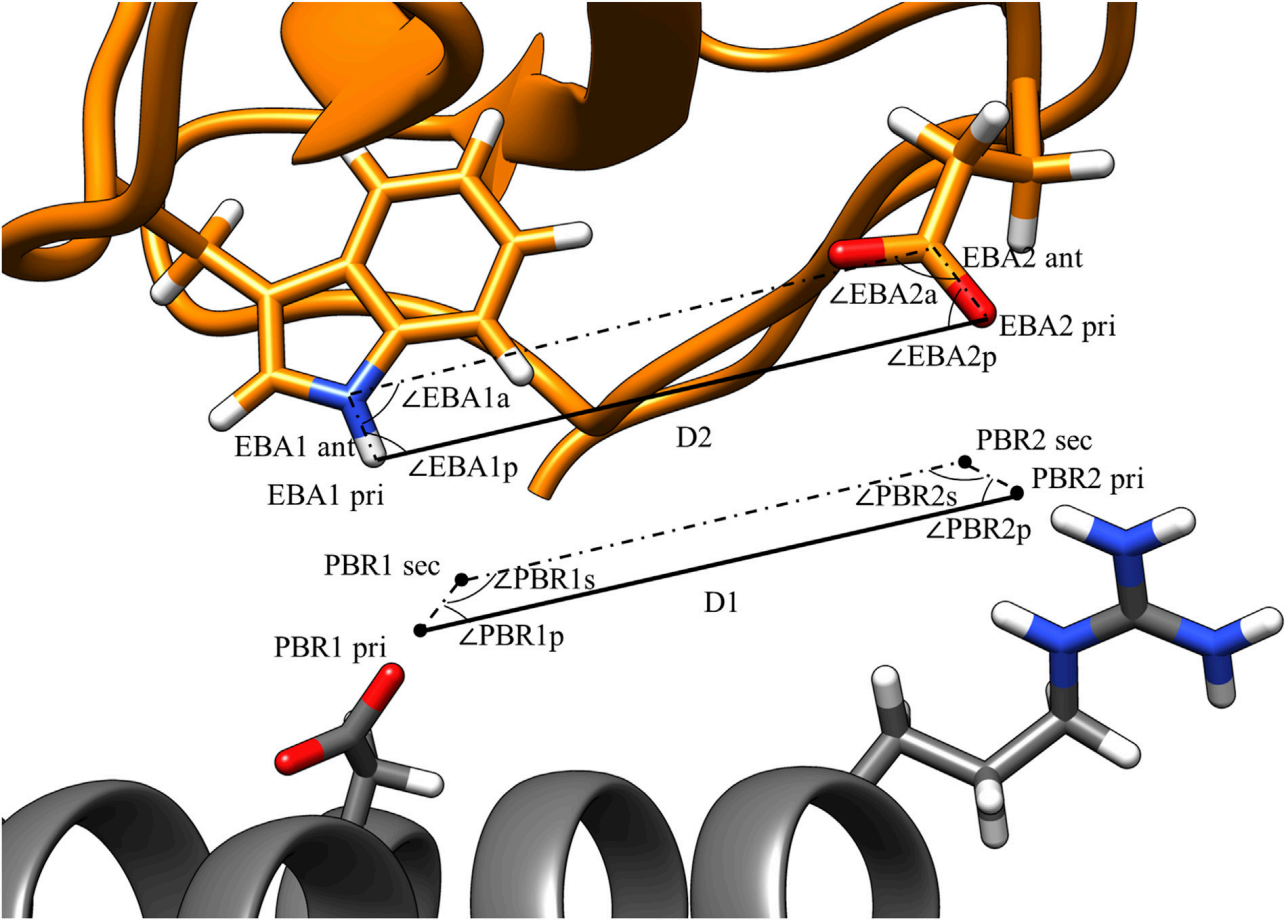
PBR and EBA pairwise distance and angle calculations. PBRs generated for paratope ASP and ARG. EBA identified for epitope TRP and GLU. The interactions being considered here are H-bonds between 1) ASP and TRP and 2) ARG and GLU. For the two interactions to be compatible, |D1 – D2| < 1.8 Å, |∠PBR1p - ∠EBA1p| < 70°, |∠PBR2p - ∠EBA2p| < 70°, |∠PBR1s - ∠EBA1a| < 70°, and |∠PBR2s - ∠EBA2a| < 70°.

Figure 3 illustrates the values that are calculated in the third step of pose identification. For each pair of PBRs, one distance and four angles are calculated. The distance is the distance between the primary points of the PBRs, while the angles are those of the quadrilateral formed by the primary and secondary points. Similar calculations are carried out for each pair of EBAs. Pairs of PBRs or EBAs that belong to the same residue are not considered in this step to encourage the formation of larger binding interfaces by MutDock. A maximum limit of one positively charged residue in the paratope is enforced in the fourth step to reduce the presence of highly flexible and unstable positively charged side chains in the interface. Thus, only one ARG/LYS residue per PBR couplet is allowed.

MutDock’s fourth step of pose identification searches for PBR and EBA sets that can coexist simultaneously. This begins by finding pairs of compatible PBR and EBA couplets. An initial screen eliminates from consideration incompatible interactions. A readily evident example would be H-bonds between ARG and ARG side chains, but many other potential interactions are also excluded from consideration. It is known that antibody binding interfaces are abundant with interactions made by pre-stabilized or low entropy side chains (Fleishman et al., 2011b). To replicate such features, H-bond type constraints are enforced to lower the chances of forming H-bonds between long chain amino acids which are unlikely to be stable. The atom and amino acid types allowed for a compatible PBR–EPA pair are listed in Table 2.

Interactions that are potentially compatible are then checked for geometric alignment using five constraints:

$$\begin{array}{c} \left|D1-D2\right|<d_{\mathrm{limit}} \end{array}$$ (1)

where D1 and D2 are the PBR and EBA distances, respectively, as calculated in the third step and d_limit_ is a user-defined threshold on the maximum permissible deviation in the primary point distances.

$$\begin{array}{c} \left|∠\mathrm{PBR}1p-∠\mathrm{EBA}1p\right|<a_{\mathrm{limit}} \end{array}$$ (2)

$$\begin{array}{c} \left|∠\mathrm{PBR}2p-∠\mathrm{EBA}2p\right|<a_{\mathrm{limit}} \end{array}$$ (3)

$$\begin{array}{c} \left|∠\mathrm{PBR}1s-∠\mathrm{EBA}1a\right|<a_{\mathrm{limit}} \end{array}$$ (4)

$$\begin{array}{c} \left|∠\mathrm{PBR}2s-∠\mathrm{EBA}2a\right|<a_{\mathrm{limit}} \end{array}$$ (5)

Geometric constraints 2–5 ensure the deviations in the interaction angles do not exceed a user-defined limit, a_limit_. For this study, d_limit_ was set at 1.8 Å, and a_limit_ was set at 70°. These values were selected as the cutoffs because they permit 85% of H-bonds from an antibody-antigen database (Chauhan et al., 2018). An illustration of these constraints is shown in Figure 3. After compatible PBR–EBA couples are found, larger sets of PBR–EBA matches are identified by searching for groups of couples that are all mutually compatible (e.g., if AB, AC, and BC are all compatible couples, then ABC must be a compatible triple). Thus, each group corresponds to a unique solution consisting of three or more predicted interface H-bonds.

In the fifth and final step of MutDock’s docking protocol, each unique solution group from the fourth step is positioned and analyzed for interface size, H-bond geometry and steric compatibility with the scaffold and antigen. The antigen is positioned so that the root-mean-square deviation (RMSD) between the primary atoms of the EBA and their corresponding PBR primary points is minimized. Next, the rotamers of the binding protein are changed to match those used in the PBRs, corresponding to the inclusion of any mutations identified during pose identification. These mutations are referred to as design mutations. If these newly placed rotamers have steric clashes with other PBR rotamers or native side chains, the pose is rejected.

Two residues are defined to have a steric clash if any of the following conditions are met: 1) Two heavy (i.e., non-hydrogen) atoms are closer than 1.3 Å, 2) More than one pair of heavy atoms are closer than 1.8 Å, or 3) One pair of heavy atoms are closer than 1.8 Å, with at least one of the two atoms being a backbone atom. Instead of calculating vdW energy between atoms as calculated using conventional approaches, MutDock uses these relaxed clash constraints under the assumption that the flexibility of the proteins would compensate for the minor steric clashes introduced in this rigid-body docking strategy. These clash definitions were selected because they consistently allowed CHARMM36 energy minimization to rectify the clashing structures, which is used as a proxy for the proteins’ flexibility.

A coarse-grained filter is used to facilitate the rejection of low-quality poses (i.e., those with small buried surface areas or major clashes between proteins). Each residue is divided into its backbone and side chain units. Each such unit is approximated as a sphere at the center of mass of the atoms with a radius equal to the distance of the farthest atom from the center of mass. Analysis of the antibody-antigen database (Chauhan et al., 2018) revealed that the coarse-grained spheres should be a minimum of 3.39 Å apart and that the complexes should have a minimum of 12 spheres in contact with one another. Poses that violate either of these requirements are rejected as having irreconcilable steric clashes or having too small interface surface areas.

After the coarse-grained filter removes obviously deficient poses, an all-atom pose validation is conducted. Each designed H-bond is accepted if the acceptor–hydrogen distance is less than 2.5 Å and the acceptor–hydrogen–donor angle is larger than 120°. Poses that fail to form even one of their predicted H-bonds are rejected. Finally, the steric compatibility between the antigen and scaffold is verified. Poses that consist of steric clashes between the antigen and the binding protein’s backbone and/or non-variable side chains are discarded, as are those that have clashes involving the residues forming the designed H-bonds. However, poses with clashes involving mutable paratope residues that do not form designed H-bonds are retained, as those residues can be changed in the second major step of MutDock: mutation.

### Mutation

Once a pose is identified, a novel feature-based approach is used to mutate variable paratope residues for one of two purposes: 1) resolving clashing variable side chains and 2) improving binding features. The mutation decisions are based on features obtained after analyzing the non-redundant database of antibody-antigen complexes. Since the approach avoids energy minimizations until the last step, the affinity maturation mutations are conservative in nature. The mutations make changes with high confidence in creating either nonpolar or polar interactions and improving binding affinity. These mutations are referred to as clash mutations.

The following steps are taken for the variable paratope residues that have steric clashes with the antigen:

1) Identify an alternate rotamer of the native amino acid that does not have steric clash with any surrounding antigen or scaffold residue.
2) If step 1 fails, search for an ASP rotamer that forms a salt bridge with an antigen residue. Salt bridges are prioritized because they are the strongest polar interactions and are less orientation dependent than H-bonds. Each antigen ARG/LYS is allowed to form a salt bridge with only one mutated paratope residue to avoid the formation of closely placed negatively charged residues.
3) If step 2 fails, search for a GLU rotamer that forms a salt bridge with an antigen residue. ASP is prioritized over GLU for salt bridge formation since it is a smaller side chain and, thus, its stabilization involves a lower entropic cost. Moreover, ASP salt bridges are more frequent than GLU salt bridges in known antibody-antigen complexes (Chauhan et al., 2018). MutDock does not consider introducing ARG/LYS mutations for salt bridge formation due to their high side chain flexibilities.
4) If step 3 fails, rotamers that can introduce new H-bonds are searched for. If a polar rotamer does not have steric clashes and forms the required type and number of H-bonds with the antigen, it is selected. Polar rotamers are searched in the sequence: SER, THR, ASN, ASP, and HIS. The required minimum number of H-bonds is one for SER and THR and two for ASP, ASN, and HIS. It was observed in the antibody-antigen database that ASN rotamers frequently make H-bonds with backbone atoms. Hence ASN rotamers making single H-bonds with antigen backbone atoms were also selected. GLN and GLU rotamers were not analyzed due to their relatively high entropy side chains.
5) If step 4 fails, rotamers that introduce nonpolar contacts are searched for. The nonpolar amino acids analyzed in this step are ALA, VAL, LEU, ILE, and PHE. A side chain atom is defined to be making a nonpolar contact if it is either a C or S atom and is less than 4.5 Å away from an antigen C or S atom. MET is not considered due to the relatively high entropy of its side chain. TRP and TYR are also not considered because they need to satisfy both polar and nonpolar requirements when buried in an interface.
6) If step 5 fails, the pose is rejected because it has an irreconcilable steric clash.

Following the mutations, the clash-free mutated poses are output in PDB format. In the current version of MutDock, no further pose refinement or ranking is performed, and rotamer repacking/energy minimization and pose ranking strategy are left to the user.

### Complex evaluation

Although force fields are not used for energy calculations during the MutDock algorithm, they were used to evaluate the MutDock predictions and compare them to other docking methods. All MutDock poses were relaxed through two energy minimization runs: CHARMM36 force field energy minimization (Vanommeslaeghe et al., 2010) with fixed backbone atoms followed by 2) Rosetta force field energy minimization (Alford et al., 2017). When docked poses are identified, they include minor steric clashes. CHARMM36 energy minimizations were able to consistently correct the clashes caused by the steric constraints used in the method, while Rosetta could only correct them sometimes. Thus, CHARMM36 was used to prepare the complexes for computational analysis while Rosetta, the most commonly used protein engineering force field, was used for comparing the quality of poses. Complexes from the previously mentioned antibody-antigen database were run through the same energy minimization routine as the MutDock poses. Since the primary purpose of MutDock is to identify high affinity mutated poses, the key metric analyzed in this work is binding energy, which is the difference in the Gibbs free energy of the system before and after complex formation. Along with computational binding energy, other binding metrics analyzed in this work, such as shape complementarity and buried interface area, were calculated using the InterfaceAnalyzer application from Rosetta (Stranges and Kuhlman, 2013).

MutDock is compared to ZDOCK 3.0.2 and HADDOCK 2.4. To compare the epitope specific/local docking of MutDock to that of ZDOCK, residues far from the epitope and paratope were manually selected to be blocked from being part of ZDOCK pose interfaces. The HADDOCK webserver was used for HADDOCK docking simulations and epitopes and paratopes were defined as the “active regions” on the webserver. For each run, each of the 2000 ZDOCK poses and 200 HADDOCK poses from the “it1” directory were run through the energy minimization routine. All protein visualization and image generation carried out done *via* UCSF Chimera (Pettersen et al., 2004).

## Results

MutDock’s performance was tested by docking 10 randomly selected antigens from the antibody-antigen database with two scaffolds: affibody and DARPin. The affibody and DARPin structures were obtained from PDB files 3MZW and 6FPA, respectively. The identities of the 13 and 18 variable residues for affibodies and DARPins were obtained from the literature (Plückthun, 2015; Ståhl et al., 2017). The paratope residues included the variable residues, as well as several surrounding residues. The epitopes from the native antibody-antigen complexes were selected as the epitopes for the docking runs. The source PDB IDs of the 10 antigen structures, residue numbers of the paratope, epitope, and variable residues are listed in Supplementary Table S1. The docking runtimes ranged from 3 to 46 h depending on the number of preliminary poses that needed to be filtered for various metrics. These high runtimes were expected, as the current implementation of MutDock is intended as a proof of principle method and its code has not been optimized for computational efficiency.

The predicted binding energies for the top poses of the 20 complexes are reported in Table 3, along with those of the native antibody-antigen complexes. Greater than 1,000 poses were identified for all antigens except PDB 3P30. That antigen consists of a two-helix bundle and hence lacks solvent-exposed backbone atoms for binding. Approximately 500 poses were identified for this antigen with both the affibody and DARPin scaffolds. The binding energies of top MutDock poses ranged from 35 to 51 kcal/mol. For eight of the ten antigens, the DARPin poses had stronger binding energies than the affibody poses. This is consistent with the fact that DARPins have larger paratopes than affibodies and our prior experience that computationally calculated binding energies are strongly correlated with interface size. The stronger calculated binding energies of the antibodies versus the MutDock-designed proteins is also consistent with this trend.

**TABLE 3:** Top binding energies (BE) of MutDock poses and their native wild type (WT) structures for the 20 antigen-scaffold complexes. The shape complementarity (Sc) values of these top MutDock poses and the native structures are also listed.

Antigen PDB	Scaffold	Top MutDock BE (kcal/mol)	Sc of top MutDock pose	WT BE (kcal/mol)	WT Sc
1JRH	Affibody	-41.31	0.64	-71.58	0.81
1JRH	DARPin	-48.67	0.61	-71.58	0.81
1OB1	Affibody	-34.95	0.53	-41.98	0.69
1OB1	DARPin	-39.54	0.59	-41.98	0.69
2XT1	Affibody	-46.83	0.74	-66.21	0.75
2XT1	DARPin	-46.38	0.66	-66.21	0.75
3BDY	Affibody	-35.07	0.60	-45.67	0.68
3BDY	DARPin	-43.44	0.56	-45.67	0.68
3L5Y	Affibody	-35.06	0.62	-54.74	0.74
3L5Y	DARPin	-42.92	0.65	-54.74	0.74
3P30	Affibody	-32.88	0.71	-32.73	0.65
3P30	DARPin	-35.11	0.50	-32.73	0.65
3X3F	Affibody	-42.95	0.64	-56.51	0.76
3X3F	DARPin	-46.56	0.59	-56.51	0.76
4AL8	Affibody	-49.89	0.61	-40.83	0.67
4AL8	DARPin	-46.66	0.70	-40.83	0.67
5DFV	Affibody	-38.38	0.56	-55.47	0.63
5DFV	DARPin	-51.02	0.56	-55.47	0.63
5IKC	Affibody	-37.87	0.62	-50.06	0.67
5IKC	DARPin	-42.75	0.61	-50.06	0.67

Table 4 lists the percentage frequencies of poses with different numbers of mutations. It is observed that the docking approach relies heavily upon design mutations for identifying poses, as more than 90% of all poses consist of either two or three design mutations. In contrast, less than 50% of all poses had any clash mutations, with a majority of them having only one mutation. Considering that all variable side chains were allowed to clash before the mutation step, this result signifies that the rotamer repacking step (i.e., step 1 of 6 of the clash mutation calculations) was efficient at resolving side chain clashes.

**TABLE 4:** Total number of poses generated and percentage frequencies of different number of design and clash mutations for each of the 20 MutDock simulations.

Antigen PDB	Scaffold	Total poses	Percentage frequency of 3 design mutations	Percentage frequency of 2 design mutations	Percentage frequency of 1 design mutation	Percentage frequency of no design mutations	Percentage frequency of any clash mutations
1JRH	Affibody	2000	41.05	54.55	4.40	0.00	47.00
1JRH	DARPin	2000	19.40	72.40	8.10	0.10	22.85
1OB1	Affibody	2000	52.85	42.90	4.25	0.00	35.10
1OB1	DARPin	2000	57.70	36.80	5.20	0.30	17.60
2XT1	Affibody	1937	71.55	25.97	2.48	0.00	39.29
2XT1	DARPin	1,654	71.28	26.36	2.36	0.00	18.38
3BDY	Affibody	1,607	70.82	26.45	2.61	0.12	43.25
3BDY	DARPin	1806	62.57	32.67	4.71	0.06	22.54
3L5Y	Affibody	2000	73.75	25.00	1.25	0.00	39.05
3L5Y	DARPin	2000	66.15	30.30	3.45	0.10	20.25
3P30	Affibody	503	70.38	26.44	2.98	0.20	34.19
3P30	DARPin	564	43.44	45.57	10.11	0.89	18.26
3X3F	Affibody	1,575	69.52	28.38	2.10	0.00	38.67
3X3F	DARPin	1,540	64.94	31.62	3.38	0.06	20.71
4AL8	Affibody	2000	64.45	31.90	3.55	0.10	39.35
4AL8	DARPin	1,120	61.61	33.04	5.27	0.09	17.95
5DFV	Affibody	2000	66.65	30.25	2.95	0.15	39.85
5DFV	DARPin	2000	67.85	29.70	2.35	0.10	16.80
5IKC	Affibody	2000	63.90	34.00	2.05	0.05	40.25
5IKC	DARPin	2000	62.55	33.80	3.50	0.15	21.65

**FIGURE 4:**
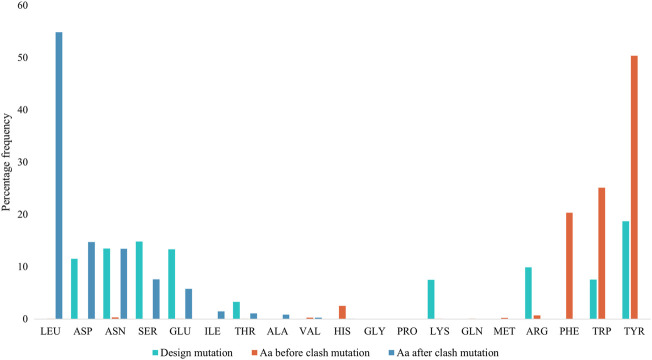
Percentage frequencies of amino acids in design mutations before and after clash mutations. In the clash mutations, aromatic amino acids which have larger side chains and lower flexibilities were mutated to smaller polar amino acids and LEU.

**FIGURE 5:**
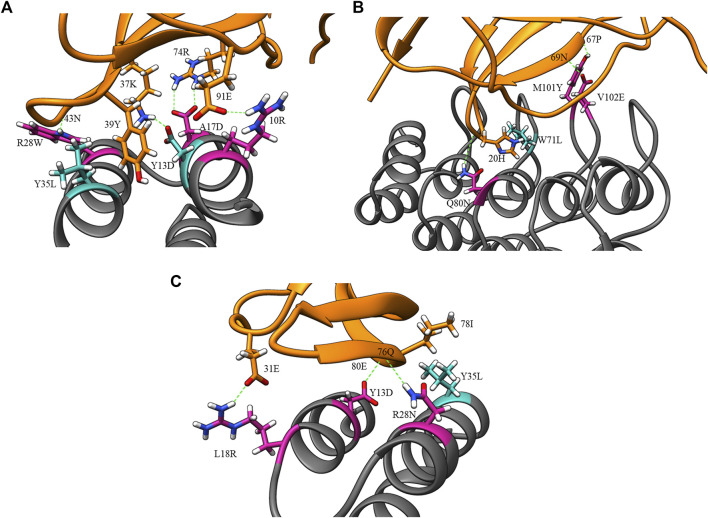
Example design and clash mutations in three MutDock designs. Design mutations are shown in pink, clash mutations are shown in dark cyan, and H-bonds are shown in broken green lines. **(A)** 1JRH-affibody. Native residue 10ARG and design mutations ARG28TRP andALA17ASP make H-bonds with 91GLU, 43ASN, and 46TRP, respectively. Clash mutation TYR13ASP makes H-bond with 37LYS. Clash mutation TYR35LEU makes hydrophobic interaction with 39TYR. **(B)** 4AL8-DARPin. Design mutations VAL102GLU, GLN80ASN, and MET101TYR make H-bonds with 69ASN, 20HIS, and 67PRO, respectively. Clash mutation TRP71LEU makes hydrophobic interaction with 20HIS. **(C)** 3BDY-affibody. Design mutations ARG28ASN, TYR13ASP, and LEU18ARG make H-bonds with 76GLN, 80GLU, and 31GLU, respectively. Clash mutation TYR35LEU makes hydrophobic interaction with 78ILE.

Figure 4 displays the percentage frequency of the amino acid types in design and clash mutations. The figure demonstrates expected trends, as MutDock favors the introduction of low entropy side chains, such as SER and TYR, that can form H-bonds with backbone atoms and other low entropy side chains. The numerical values for Figure 4 are listed in Supplementary Table S2. In contrast, the positively charged, high entropy side chains of ARG and LYS are disfavored. The most favored clash mutation was LEU. Examples of design and clash mutations are illustrated in Figure 5.

The widely used docking programs ZDOCK and HADDOCK were tested on the same affibody and DARPin scaffolds against the same antigens. ZDOCK was selected for comparison with MutDock since neither method uses local pose refinement or rigid body energy minimizations (Pierce et al., 2014).

**FIGURE 6:**
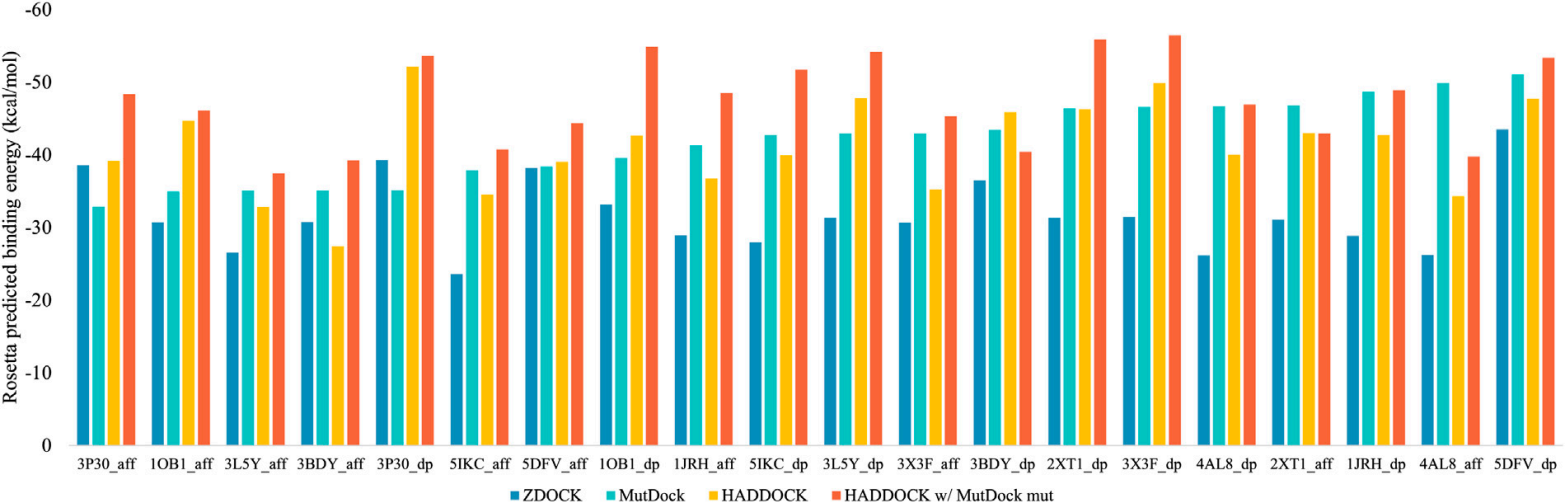
Top Rosetta-predicted computational binding energies of poses from the 20 docking simulations performed using ZDOCK, MutDock, HADDOCK, and a combination approach of HADDOCK with top MutDock scaffold.

The top binding energies for each complex, along with the difference with the best MutDock pose energies, are displayed in Figure 6. Supplementary Table S4 lists these top binding energy values along with their differences. A more negative predicted binding energy value corresponds to a higher likelihood of binding, and it is typical for computational predictions to have larger magnitudes than experimental values. MutDock predicted poses with binding energies at least 3 kcal/mol stronger than ZDOCK in 17 of 20 complexes and at least 10 kcal/mol stronger in 11 complexes. The only antigen ZDOCK outperformed MutDock on was 3P30, whose helical nature eliminates the possibility of the backbone H-bonds that MutDock preferentially targets.

MutDock was also compared to HADDOCK (van Zundert et al., 2016; Honorato et al., 2021), a docking approach that performs local rigid body energy minimizations along with further refinement using short MD simulations. The top binding energies for each complex are displayed in Figure 6. MutDock predicted better poses for nine complexes, similar quality poses (i.e., ±3 kcal/mol) in five complexes, and worse poses for six complexes. In the nine complexes where MutDock outperformed HADDOCK, the improvement in binding energies was lower than those obtained from the ZDOCK comparisons. Thus, HADDOCK predicted complexes with stronger binding energies than ZDOCK.

A likely cause of HADDOCK’s performance being evaluated well by energy calculations is its use of such calculations to refine initial poses. To investigate whether the positional refinements HADDOCK utilizes could further improve the MutDock poses, the top MutDock designs were docked using HADDOCK. After HADDOCK docking, poses were defined as near-native if they had an interface Cα RMSD less than 4 Å when compared with the respective top MutDock pose.

Figure 6 also displays the top binding energies from the HADDOCK and MutDock combination approach. Supplementary Table S4 lists these binding energies along with the binding energies of near-native poses, their RMSDs and the respective binding energy differences. For nine complexes, HADDOCK identified novel poses with better binding energies than the native MutDock poses. Of those nine complexes, HADDOCK identified no near-native pose for two complexes, better (i.e., by at least 3 kcal/mol) poses for three complexes, comparable poses for two complexes, and worse than native poses for two complexes. For the remaining eleven complexes, HADDOCK identified better poses for two complexes, comparable poses for six complexes, and worse poses for three complexes. Finally, the top HADDOCK poses using the MutDock designed binding proteins had stronger calculated binding energies for 16/20 complexes than the top HADDOCK poses with the original scaffolds.

## Discussion

MutDock is a novel computational approach for the generation of mutated, docked scaffolds designed to bind target epitopes. The approach identifies regions around the scaffold paratope which can host polar epitope atoms to form H-bonds. Pairwise distance alignment between the epitope atoms and H-bond regions is used to obtain groups of low entropy H-bonds that can be formed simultaneously. Each group constitutes a unique pose that is passed through several compatibility filters. MutDock was benchmarked by docking ten antigens with two scaffolds. The predicted binding energies of the top MutDock poses were comparable to those of known binding complexes when accounting for the influence of interface size on calculated energies. The MutDock poses were significantly better than ZDOCK’s results for 17 of the 20 predicted complexes. When compared to HADDOCK, MutDock performed better in 9/20 complexes, comparably in 5/20, and worse in the remaining 6/20 complexes. However, the HADDOCK scores improved for 16/20 complexes when HADDOCK was used to dock the MutDock-designed scaffolds.

It is particularly notable that more than 90% of the poses MutDock identified required at least two mutations. This shows that MutDock has the ability to generate a large number of poses with multiple beneficial mutations per pose. Conventional design approaches search for beneficial mutations through iterative cycles of random point mutations (Pantazes et al., 2015; Adolf-Bryfogle et al., 2018). On the other hand, MutDock is able to simultaneously identify multiple beneficial mutations per pose in a single search step, with further beneficial mutations added in the clash-removal step. Such an approach allows MutDock to search a larger solution sequence space and hence identify poses that would not be identified by fixed-sequence docking methods.

Using Rosetta-calculated binding energies as a benchmark, MutDock performed significantly better than ZDOCK for most of the complexes. Each of these methods relies on geometric criteria for identifying binding poses: H-bond formation for MutDock and shape complementarity along with molecular mechanics for ZDOCK. This is in line with expectations, as the mutations introduced by MutDock should result in improved binding energies relative to those attainable by the original scaffold. Nonetheless, this demonstrates that by strictly using geometric criteria, MutDock is able to identify favorable and promising binding conformations.

The comparison of the performances of MutDock and HADDOCK is more nuanced. Each did best on approximately half of the complexes in a head-to-head comparison. This is due in part to the fact that each has an advantage over the other: MutDock allows for mutation of the scaffold, while HADDOCK uses energy minimizations and positional refinement to maximize predicted binding energies. However, 16/20 complexes were improved when HADDOCK used the top MutDock-designed scaffolds compared to when it used the original ones, albeit not always in conformations similar to MutDock’s predicted poses. This indicates that MutDock’s predicted mutations, which are unguided by energy calculations, create the potential to improve binding energies. It is notable that in a number of complexes, HADDOCK was unable to identify poses with binding energies as strong as MutDock’s predictions. Given that it is demonstrable that those poses exist, this indicates that HADDOCK’s energy-based pose identification algorithm still has potential room for improvement.

One of the primary motivations of MutDock was to explore the design of binding proteins without placing the ligand protein around the receptor in a random or grid-based position. Docking tools such as HADDOCK obtain initial poses through random starting orientations and refine these poses via forcefield-dependent energy minimizations, while MutDock only generates poses that consist of the required minimum number of H-bonds. We believe that replicating known structural or conformational features *via* simple geometric alignments has the potential to identify superior poses compared to using random or grid-based initial poses. Despite only targeting H-bonds in the binding interface, MutDock was able to generate poses with computational metrics comparable to known binding complexes and poses made with other docking programs. Thus, MutDock serves as an example of a viable docking-design approach that attempts to replicate known beneficial features of binding interfaces, such as hotspot interactions in hotspot-centric design (Fleishman et al., 2011a). The development of such methods has been made possible by the availability of large datasets of known complex structures that can be analyzed for common key structural features which can later be targeted.

The only other interaction-based docking approach we have seen in the literature is RIFdock (Dou et al., 2018; Longxing et al., 2020). Compared to MutDock, RIFdock uses a larger library of rotamer poses and includes hydrophobic interactions as target interactions too. A major difference between the two approaches is the search strategy. RIFdock moves the receptor protein in 6D steps, with increasing resolution, around the target protein to find scaffold poses that host multiple rotamers, which make strong interactions with the epitope. On the other hand, MutDock uses pairwise distance alignment to identify groups of compatible interactions in one step. However, since a detailed description of the RIFdock methodology is not available in a peer-reviewed article, a more thorough comparison of the approaches was not possible.

We plan to make several improvements to the current MutDock implementation. ZDOCK run times lasted for 4 min while HADDOCK webservers took a maximum of approximately 6 h, which also includes the time when the job was queued, for each docking run. On the other hand, MutDock runtimes ranged from 3 to 46 h on a 3.00 GHz Intel Xeon Gold 6248R processor. To reduce these run times, we plan on optimizing and shifting the code from Cython to C++. Hydrophobic interactions are as important as electrostatic interactions in protein-protein binding (Wang et al., 2018). However, targeting their formation is challenging with the current MutDock thresholds since hydrophobic interactions are not as geometrically constrained as H-bonds. Similar to RIFdock, we plan to modify the approach to target hydrophobic interactions with a more robust rotamer library. We expect MutDock to generate better quality poses in smaller run times after these modifications are made.

The recent breakthroughs of AlphaFold and RosettaFold at predicting protein structures without heavy reliance on physics-based force fields herald a change in computational protein engineering. We believe that one of the frontiers of bioengineering will be the growth of computational protein design methods that use machine learning and engineering principles instead of force fields. MutDock demonstrates the potential of such approaches. Through spatial positioning and mutation steps, MutDock is able to identify poses that have many low entropy and favorable interactions. The results, especially those from re-docking the scaffolds with HADDOCK, indicate that the binding energies can be improved without relying on force field calculations. The MutDock code and all data from this project have been uploaded to the Dryad Data Repository and are available for free public access at https://doi.org/10.5061/dryad.0rxwdbs3q.

## Data Availability

All original contributions, data, and code presented in the study are included in the article, included in the Supplementary Material, or have been uploaded to the Dryad Data Repository and are available for free public access at https://doi.org/10.5061/dryad.0rxwdbs3q. Further inquiries can be directed to the corresponding author.
