# Supplementary material for: MutDock: A computational docking approach for fixed-backbone protein scaffold design
Source: Front Mol Biosci. 2022 Aug 29;9:933400. doi: 10.3389/fmolb.2022.933400 (PMC9465448; doi:10.3389/fmolb.2022.933400)
Supplement: Supplementary file 1 [file DataSheet1.docx]

Supplementary Material

# Supplementary Table

Table 1 Residue numbers of “active binding regions” in HADDOCK simulations. Residue numbers of epitope, paratope and variable paratope residue numbers for MutDock runs is also provided. For 4AL8-dp and 1OB1-dp MutDock simulations, the number of epitope residues were reduced to increase runtimes.

| Complex | Residues |
| --- | --- |
|  | HADDOCK |
| 1JRH | 37, 38, 39, 40, 41, 42, 43, 44, 45, 46, 66, 67, 68, 69, 70, 72, 74, 88, 89, 90, 91, 93 |
| 1OB1 | 1, 6, 7, 8, 9, 10, 11, 12, 13, 14, 15, 16, 18, 23, 24, 25, 26, 28, 31, 36, 38, 39, 40 |
| 2XT1 | 1, 2, 3, 4, 5, 6, 7, 9, 26, 30, 31, 32, 33, 35, 36, 39, 40, 42, 43, 44, 47, 49 |
| 3BDY | 31, 32, 35, 66, 68, 69, 70, 71, 72, 73, 74, 75, 76, 77, 78, 79, 81 |
| 3L5Y | 1, 2, 3, 6, 7, 9, 10, 13, 74, 75, 76, 78, 79, 81, 82, 83, 85 |
| 3P30 | 52, 55, 56, 59, 60, 62, 63, 64, 66, 67, 69, 70, 71, 73, 74, 77 |
| 3X3F | 14, 15, 16, 17, 18, 19, 28, 29, 33, 35, 36, 37, 38, 39, 40, 41, 42 |
| 4AL8 | 13, 14, 16, 17, 18, 19, 20, 21, 22, 24, 26, 52, 54, 55, 56, 57, 58, 66, 67, 68, 69, 71 |
| 5DFV | 13, 17, 20, 21, 22, 23, 24, 25, 26, 27, 28, 32, 49, 50, 51, 52, 53, 55, 56, 57, 58, 59, 60, 69 |
| 5IKC | 9, 10, 11, 12, 13, 14, 15, 16, 43, 44, 45, 46, 47, 48, 80, 82, 83, 84, 85, 87 |
| Darpin | 3, 4, 5, 6, 7, 8, 9, 10, 11, 12, 13, 14, 33, 34, 35, 36, 37, 38, 39, 40, 41, 42, 43, 44, 45, 46, 47, 68, 69, 70, 71, 72, 73, 74, 75, 76, 77, 78, 79, 80, 101, 102, 103, 104, 105, 106, 107, 108, 109, 110, 111, 112, 113, 135, 136, 137, 138, 139, 140, 141, 142, 143, 144, 145, 146, 147, 148 |
| Affibody | 6, 7, 9, 10, 11, 13, 14, 15, 16, 17, 18, 23, 24, 25, 27, 28, 29, 31, 32, 34, 35, 36, 37 |
|  | MutDock |
| 1JRH_aff | 37, 38, 39, 40, 41, 42, 43, 44, 45, 46, 66, 67, 68, 69, 70, 72, 74, 88, 89, 90, 91, 93 |
| 1JRH_dp | 37, 38, 39, 40, 41, 42, 43, 44, 45, 46, 66, 67, 68, 69, 70, 72, 74, 88, 89, 90, 91, 93 |
| 1OB1_aff | 1, 6, 7, 8, 9, 10, 11, 12, 13, 14, 15, 16, 18, 23, 24, 25, 26, 28, 31, 36, 38, 39, 40 |
| 1OB1_dp | 8, 9, 10, 11, 13, 14, 15, 16, 23, 24, 25, 26, 28, 36, 38, 39, 40 |
| 2XT1_aff | 1, 2, 3, 4, 5, 6, 7, 9, 26, 30, 31, 32, 33, 35, 36, 39, 40, 42, 43, 44, 47, 48 |
| 2XT1_dp | 1, 2, 3, 4, 5, 6, 7, 9, 26, 30, 31, 32, 33, 35, 36, 39, 40, 42, 43, 44, 47, 48 |
| 3BDY_aff | 31, 32, 35, 66, 68, 69, 70, 71, 72, 73, 74, 75, 76, 77, 78, 79, 80 |
| 3BDY_dp | 31, 32, 35, 66, 68, 69, 70, 71, 72, 73, 74, 75, 76, 77, 78, 79, 80 |
| 3L5Y_aff | 1, 2, 3, 6, 7, 9, 10, 13, 74, 75, 76, 78, 79, 81, 82, 83, 84 |
| 3L5Y_dp | 1, 2, 3, 6, 7, 9, 10, 13, 74, 75, 76, 78, 79, 81, 82, 83, 84 |
| 3P30_aff | 55, 56, 59, 60, 62, 63, 64, 66, 67, 69, 70, 71, 73, 74, 77 |
| 3P30_dp | 55, 56, 59, 60, 62, 63, 64, 66, 67, 69, 70, 71, 73, 74, 77 |
| 3X3F_aff | 6, 14, 16, 17, 18, 19, 29, 33, 35, 36, 37, 38, 39, 40, 41, 42 |
| 3X3F_dp | 6, 14, 16, 17, 18, 19, 29, 33, 35, 36, 37, 38, 39, 40, 41, 42 |
| 4AL8_aff | 13, 14, 16, 17, 18, 19, 20, 21, 22, 24, 26, 52, 54, 55, 56, 57, 58, 66, 67, 68, 69, 71 |
| 4AL8_dp | 13, 14, 17, 18, 19, 20, 21, 22, 26, 52, 54, 56, 66, 67, 68, 69, 71 |
| 5DFV_aff | 13, 17, 20, 21, 22, 23, 24, 25, 26, 27, 28, 32, 49, 50, 51, 52, 53, 55, 56, 57, 58, 59, 60, 68 |
| 5DFV_dp | 13, 17, 20, 21, 22, 23, 24, 25, 26, 27, 28, 32, 49, 50, 51, 52, 53, 55, 56, 57, 58, 59, 60, 68 |
| 5IKC_aff | 9, 10, 11, 12, 13, 14, 15, 16, 43, 44, 45, 46, 47, 48, 80, 82, 83, 84, 85, 86 |
| 5IKC_dp | 9, 10, 11, 12, 13, 14, 15, 16, 43, 44, 45, 46, 47, 48, 80, 82, 83, 84, 85, 86 |
| Darpin | 6, 7, 10, 14, 15, 16, 33, 34, 35, 36, 37, 38, 43, 46, 47, 66, 68, 69, 70, 71, 79, 80, 99, 101, 102, 103, 104, 112, 113, 132, 134, 135, 136, 137, 145, 146, 147 |
| Affibody | 6, 9, 10, 11, 13, 14, 15, 17, 18, 24, 25, 27, 28, 31, 32, 35, 36, 37, 38 |
|  | MuDock variable residues |
| Affibody | 9, 10, 11, 13, 14, 17, 18, 24, 25, 27, 28, 32, 35 |
| Darpin | 33, 35, 36, 38, 46, 47, 66, 68, 69, 71, 79, 80, 99, 101, 102, 104, 112, 113 |

Table 2 Percentage frequencies of different amino acids in design mutations, before and after clash mutations in all 20 MutDock simulations.

| **Amino acid** | **Percentage frequency in design mutations** | **Percentage frequency in residues before clash mutation** | **Percentage frequency in residues after clash mutations** |
| --- | --- | --- | --- |
| ARG | 9.86 | 0.70 | 0.00 |
| ALA | 0.00 | 0.00 | 0.81 |
| ASN | 13.48 | 0.29 | 13.41 |
| ASP | 11.51 | 0.00 | 14.74 |
| GLN | 0.00 | 0.05 | 0.00 |
| GLU | 13.34 | 0.06 | 5.78 |
| GLY | 0.00 | 0.00 | 0.00 |
| HIS | 0.00 | 2.49 | 0.03 |
| ILE | 0.00 | 0.00 | 1.46 |
| LEU | 0.00 | 0.07 | 54.89 |
| LYS | 7.48 | 0.02 | 0.00 |
| MET | 0.00 | 0.20 | 0.00 |
| PHE | 0.00 | 20.33 | 0.00 |
| PRO | 0.00 | 0.00 | 0.00 |
| SER | 14.82 | 0.00 | 7.58 |
| THR | 3.28 | 0.03 | 1.08 |
| TRP | 7.55 | 25.14 | 0.00 |
| TYR | 18.69 | 50.37 | 0.00 |
| VAL | 0.00 | 0.25 | 0.23 |

Table 3 Top binding energies (BE) from the MutDock and ZDOCK docking simulations along with their energy differences for the 20 antigen-scaffold complexes.

| **Antigen PDB** | **Scaffold** | **Top MutDock BE (kcal/mol)** | **Top ZDOCK BE (kcal/mol)** | **Top ZDOCK BE - Top MutDock BE (kcal/mol)** |
| --- | --- | --- | --- | --- |
| 1JRH | Affibody | -41.3 | -28.9 | 12.4 |
| 1JRH | DARPin | -48.7 | -28.8 | 19.8 |
| 1OB1 | Affibody | -35.0 | -30.7 | 4.2 |
| 1OB1 | DARPin | -39.5 | -33.2 | 6.4 |
| 2XT1 | Affibody | -46.8 | -31.1 | 15.8 |
| 2XT1 | DARPin | -46.4 | -31.3 | 15.0 |
| 3BDY | Affibody | -35.1 | -30.8 | 4.3 |
| 3BDY | DARPin | -43.4 | -36.5 | 7.0 |
| 3L5Y | Affibody | -35.1 | -26.5 | 8.6 |
| 3L5Y | DARPin | -42.9 | -31.3 | 11.6 |
| 3P30 | Affibody | -32.9 | -38.6 | -5.7 |
| 3P30 | DARPin | -35.1 | -39.3 | -4.2 |
| 3X3F | Affibody | -43.0 | -30.7 | 12.3 |
| 3X3F | DARPin | -46.6 | -31.4 | 15.1 |
| 4AL8 | Affibody | -49.9 | -26.2 | 23.7 |
| 4AL8 | DARPin | -46.7 | -26.1 | 20.5 |
| 5DFV | Affibody | -38.4 | -38.2 | 0.2 |
| 5DFV | DARPin | -51.0 | -43.5 | 7.5 |
| 5IKC | Affibody | -37.9 | -23.6 | 14.3 |
| 5IKC | DARPin | -42.8 | -28.0 | 14.8 |

Table 4 Top binding energies (BE) from the MutDock and HADDOCK with native and MutDock scaffold docking simulations along with their energy differences for the 20 antigen-scaffold complexes. Also listed are the best binding energies of near native poses from the HADDOCK docking with MutDock scaffolds and the RMSD of these poses. All binding energies are reported in kcal/mol.

| **Antigen PDB** | **Scaffold** | **Top HADDOCK BE** | **Top HADDOCK BE - Top MutDock BE** | **Top HADDOCK w/ MutDock scaffold (HAD-Mut) BE** | **Top HADDOCK BE - Top HAD-Mut BE** | **Top near native BE** | **RMSD (Å)** | **Top MutDock BE - Top HAD-Mut BE** |
| --- | --- | --- | --- | --- | --- | --- | --- | --- |
| 1JRH | Affibody | -36.7 | 4.6 | -48.5 | 11.8 | -48.5 | 0.7 | 7.2 |
| 1JRH | DARPin | -42.7 | 6.0 | -48.9 | 6.2 | -41.5 | 1.8 | 0.2 |
| 1OB1 | Affibody | -44.7 | -9.7 | -46.1 | 1.5 | None | None | 11.2 |
| 1OB1 | DARPin | -42.6 | -3.1 | -54.9 | 12.3 | -45.2 | 0.6 | 15.3 |
| 2XT1 | Affibody | -43.0 | 3.9 | -42.9 | -0.1 | -42.9 | 0.7 | -3.9 |
| 2XT1 | DARPin | -46.3 | 0.1 | -55.9 | 9.6 | -41.7 | 3.0 | 9.5 |
| 3BDY | Affibody | -27.4 | 7.7 | -39.2 | 11.8 | -35.0 | 0.6 | 4.2 |
| 3BDY | DARPin | -45.9 | -2.4 | -40.4 | -5.5 | None | None | -3.0 |
| 3L5Y | Affibody | -32.8 | 2.2 | -37.4 | 4.6 | -37.4 | 1.5 | 2.4 |
| 3L5Y | DARPin | -47.8 | -4.9 | -54.2 | 6.3 | -41.9 | 2.4 | 11.3 |
| 3P30 | Affibody | -39.1 | -6.3 | -48.4 | 9.2 | -27.5 | 3.7 | 15.5 |
| 3P30 | DARPin | -52.1 | -17.0 | -53.6 | 1.5 | None | None | 18.5 |
| 3X3F | Affibody | -35.3 | 7.7 | -45.3 | 10.0 | None | None | 2.3 |
| 3X3F | DARPin | -49.9 | -3.4 | -56.5 | 6.6 | -50.7 | 1.0 | 9.9 |
| 4AL8 | Affibody | -34.3 | 15.6 | -39.7 | 5.4 | -39.7 | 0.7 | -10.2 |
| 4AL8 | DARPin | -40.0 | 6.6 | -46.9 | 6.9 | -46.9 | 0.6 | 0.3 |
| 5DFV | Affibody | -39.0 | -0.7 | -44.3 | 5.3 | -41.9 | 3.1 | 6.0 |
| 5DFV | DARPin | -47.7 | 3.3 | -53.4 | 5.7 | -53.4 | 1.1 | 2.4 |
| 5IKC | Affibody | -34.6 | 3.3 | -40.7 | 6.2 | -34.0 | 2.4 | 2.9 |
| 5IKC | DARPin | -40.0 | 2.8 | -51.7 | 11.8 | -49.6 | 3.4 | 9.0 |
